# The Retroacetabular Angle Determines the Safe Angle for Screw Placement in Posterior Acetabular Fracture Fixation

**DOI:** 10.1155/2013/432675

**Published:** 2013-05-28

**Authors:** Ayman M. A. Tadros, Thomas R. Oxland, Peter O'Brien

**Affiliations:** ^1^Trauma Division, Orthopaedic Department, University of BC, British Columbia, Canada; ^2^Orthopaedic Department, Mafraq Hospital, Abu Dhabi, UAE; ^3^Division of Orthopaedic Engineering Research, Departments of Orthopaedics and Mechanical Engineering, University of British Columbia, BC, Canada

## Abstract

*Introduction*. A method for the determination of safe angles for screws placed in the posterior acetabular wall based on preoperative computed tomography (CT) is described. It defines a retroacetabular angle and determines its variation in the population. *Methods*. The retroacetabular angle is the angle between the retroacetabular surface and the tangent to the posterior acetabular articular surface. Screws placed through the marginal posterior wall at an angle equal to the retroacetabular angle are extraarticular. Medial screws can be placed at larger angles whose difference from the retroacetabular angle is defined as the allowance angles. CT scans of all patients with acetabular fractures treated in our institute between September 2002 to July 2007 were used to measure the retroacetabular angle and tangent. *Results*. Two hundred thirty one patients were included. The average (range) age was 42 (15–74) years. The average (range) retroacetabular angle was 39 (30–47) degrees. The average (range) retroacetabular tangent was 36 (30–45) mm. *Conclusions*. Placing the screws at an average (range) angle of 39 (33–47) degrees of anterior inclination with the retroacetabular surface makes them extraarticular. Angles for medial screws are larger. Safe angles can be calculated preoperatively with a computer program.

## 1. Introduction

Fractures involving the posterior acetabular wall and column are the most common acetabular fractures [1–3]. The midportion of the posterior acetabulum above the ischial spine was considered by Matta et al. to be the danger zone of the acetabulum [4], a fact which was emphasized by more recent anatomical studies [5]. Some authors advised avoiding insertion of screws in the danger zone to avoid the negative consequence of intraarticular screw penetration [6]. The latter is a recognized complication of acetabular fracture surgery [7, 8]. Ebraheim et al. verified the boundaries of the danger zone by a cadaver study, and they recommended that a screw placed at an angle of 30–49 degrees of medial angulation to the perpendicular of the posterior wall should be extraarticular [9]. They measured this angle at one centimeter medial to the posterior margin of the acetabulum. Consequently, the safe angle for fixing marginal posterior wall fractures cannot be predicted depending on their study. Bosse advised that placing a screw in the coronal plane perpendicular to the long axis of the patient's body would be extraarticular [10]. However, placing all posterior wall screws in the plane defined by Bosse [10] might compromise the stability of fracture fixation. The screws in the medial part of the posterior wall can be placed more perpendicular to the retroacetabular surface giving more stable fracture fixation. 

Screw trajectories can be determined using preoperative computed tomography (CT) scan of the pelvis [11–15]. The latter is supported by various computer software which creates either fracture templates [11] or virtual reality simulation [13–15]. However, all those tools aim at supporting the preoperative surgical planning rather than execution of a surgical technique [15].

The aim of this study is to describe a method for the determination of safe angles for screws placed in the posterior acetabular wall using preoperative CT scan of the pelvis. The method involves defining a retroacetabular angle, determining its variation in the population sector, that is, vulnerable to acetabular fractures, and presentating a simple computer-based tool for the accurate preoperative calculation of safe angles for placement of screws in the posterior acetabular wall. 

## 2. Material and Methods

### 2.1. Rationale

**Figure 1:**
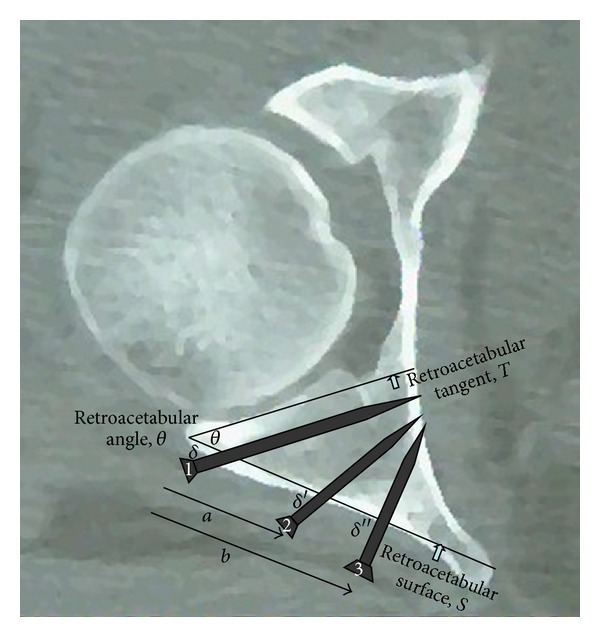
Diagrammatic illustration showing the retroacetabular surface (*S*) and the retroacetabular tangent (*T*) with the retroacetabular angle in between (*θ*). The latter is geometrically equivalent to angle *δ* which is a safe angle for placing screw 1 at the lateral margin of the posterior wall of the acetabulum. Screw 1 is parallel to the retroacetabular tangent and would not penetrate the joint. Screws 2 and 3 that are placed at distances *a* and *b*, respectively, from the margin of the posterior wall (medialization distance) can be inserted safely at bigger angles than the retroacetabular angle (*δ*′ and *δ*′′).

 The posterior articular surface of the acetabulum makes an angle with the retroacetabular surface (*S* in Figure 1). We named this angle the retroacetabular angle (*θ* in Figure 1), which we defined as the angle between the retroacetabular surface and a line drawn tangential to the posterior articular surface. We called that line the retroacetabular tangent (*T* in Figure 1). A screw inserted through the posterior acetabular wall that runs parallel to the retroacetabular tangent avoids articular penetration (screw 1 in Figure 1). The angle between that screw and the retroacetabular surface (*δ* in Figure 1) is geometrically equal to the retroacetabular angle. A screw angle equivalent to the retroacetabular angle should be a safe angle for screw placement at the lateral margin of the posterior wall of the acetabulum. Screws that are more medial along the posterior wall (screws 2 and 3 in Figure 1) can be placed safely at angles that are larger than the retroacetabular angle (*δ*′, *δ*′′ in Figure 1). The increment in their placement angle above the magnitude of the retroacetabular angle was termed the allowance angle, and the distance from the posterior acetabular margin to their site of insertion was termed the medialization distance (distances *a* and *b* in Figure 1).

**Figure 2:**
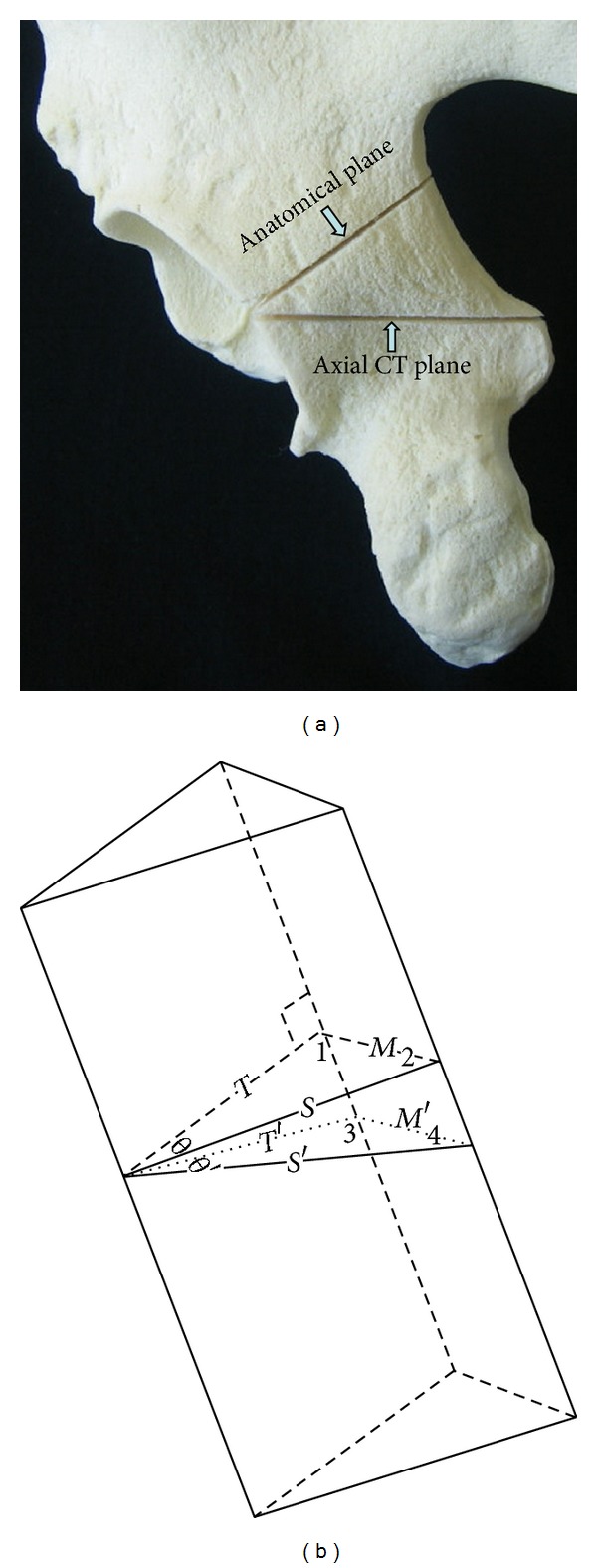
(a) Pelvic model showing the oblique plane of the axial CT scan cuts which is used for preoperative calculation of safe screw angles and the anatomical plane perpendicular to the acetabulum. The latter is the usual plane of screw placement. (b) Diagrammatic illustration showing the posterior wall as a three-sided prism with the two planes described in (a). Due to the obliquity of the CT plane, *S*′and *T*′are longer than *S* and *T*. This makes angles 3 and 4 in the oblique plane bigger than angles 1 and 2 in the perpendicular plane. Since *M* equals *M*′ angle *θ*′ which represents the retroacetabular angle in CT cuts and is used in calculation of safe screw angles is smaller than *θ* which represents the retroacetabular angle measured in a plane perpendicular to the acetabulum. The retroacetabular angle measured on CT is thus a safer angle.

Due to the cephalocaudal acetabular convergence, conventional axial CT cuts pass through the acetabulum at an oblique plane. The retroacetabular angle in pelvis trauma CT scan appears smaller than that in the anatomical cuts, perpendicular to the acetabulum (Figure 2). Consequently, calculations based on the CT scans are conservative for determining safe angles for screws, which are usually placed across the anatomical plane.

### 2.2. Technique for Preoperative Planning

A trauma pelvis CT scan is obtained for all patients with acetabular fractures. The axial CT cuts of the nonfractured contralateral acetabulum at the level of the fracture are used for preoperative planning. Fracture lines are drawn on the posterior wall of the intact acetabulum to represent the fracture fragments after reduction, and the sites of screws needed for fixation are determined. The retroacetabular angle, the length of the retroacetabular tangent, and the medialization distance for the site of screw insertion are measured.

**Figure 3:**
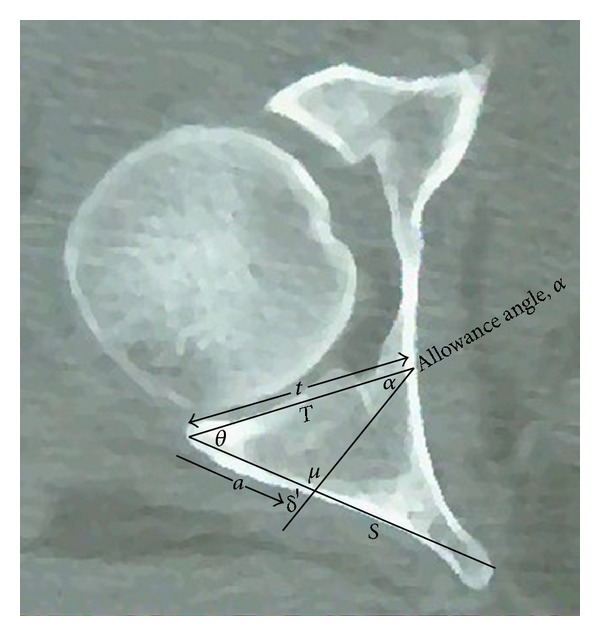
Diagrammatic illustration showing that the screw angle *δ*′ for a screw placed at a medialization distance a should equal the sum of retroacetabular angle *θ* and the allowance angle *α*.

A screw placed at the posterior acetabular margin should be outside the joint if inserted through the retroacetabular surface at an angle that equals the retroacetabular angle. For more medially placed screws, the angle of their placement (*δ*′ in Figure 3) should equal the sum of the retroacetabular angle (*θ* in Figure 3) and the allowance angle (*α* in Figure 3). The allowance angle can be calculated as follows. Providing the retroacetabular angle as *θ*, the allowance angle as *α*, the screw angle as *δ*′, the angle opposite the retroacetabular acetabular tangent as *μ*, the length of the retroacetabular acetabular tangent is *t* and the medialization distance as *a* (Figure 3) and all angles measured in degrees and all lengths in mm, the allowance angle is calculated from the following equation:

(1) $$\begin{array}{c} \tan\alpha =\frac{a\sin\theta }{t-a\cos\theta }. \end{array}$$

The equation above is derived as follows. First, given the triangle shown in Figure 3, we know that

(2) $$\begin{array}{c} \mu =180-\left(\theta +\alpha \right). \end{array}$$

Using the law of sines, we can write

(3) $$\begin{array}{c} \frac{a}{\sin\alpha }=\frac{t}{\sin\mu }. \end{array}$$

Incorporating (2) into (3), we have

(4) $$\begin{array}{c} \frac{\sin\left(180-\left(\theta +\alpha \right)\right)}{\sin\alpha }=\frac{t}{a}. \end{array}$$

It can be shown that

(5) $$\begin{array}{c} \sin\left(180-\left(\theta +\alpha \right)\right)=\sin\theta \cos\alpha +\cos\theta \sin\alpha . \end{array}$$

Substituting (5) into (4) and simplifying yields

(6) $$\begin{array}{c} \frac{\sin\theta }{\tan\alpha }+\cos\theta =\frac{t}{a}. \end{array}$$

Further simplification of (6) yields (1), our relationship for the allowance angle, *α*, in terms of the retroacetabular angle, *θ*, and the distances *t* and *a*. 

### 2.3. Patients and Methods

Demographic data of a consecutive series of trauma patients with acetabular fractures treated at our institution, which is a level-one trauma centre, between September 2002 and July 2007 were identified from a prospectively collected trauma database. The CT scans of the patients were retrieved from the picture archiving and communication system (PACS) (iSite version 3.3.2 Release (Build 4) FCS, Stentor Intelligent Informatics, Philips, California, USA). The axial CT scans of all patients were reviewed. All patients had the same trauma CT protocol adopted with 3-mm overlapping sections. Patients with manifestations of advanced osteoarthritis of their hips were excluded from the study. The nonfractured acetabulum was used to measure the retroacetabular angle and the retroacetabular tangent at the midportion of the posterior acetabular wall. The measurements were executed using the computer software of the PACS system. 

Computer software (Excel, Microsoft Office, version 2003, Microsoft, USA) was used to develop a spread sheet that can be used to calculate the allowance angles corresponding to variable retroacetabular angles and tangents for every one mm of medialization distance of screw site. 

This study was approved by the research ethical committee of our institution.

## 3. Results

**Figure 4:**
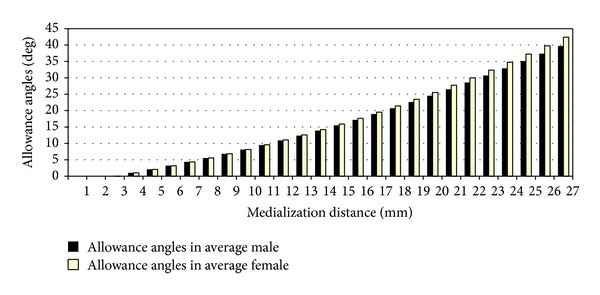
Graphic representation of the increments of the allowance angle for screws placed more medially in the posterior acetabular wall for the average retroacetabular angle and tangent in both male and female patients.

Two hundred forty two patients with acetabular fracture were retrieved from the trauma database. Eleven patients were excluded from the study due to the presence of advanced osteoarthritis of the hip. The remaining 231 patients were included in the study. One hundred seventy eight were males and 53 were females. The average (range) age was 42 (15–74) years. The average (range) retroacetabular angle was 40 (33–47) degrees for men and 37 (30–40) degrees for women (*P* = 0.000012, *t*-test). The average (range) retroacetabular tangent was 37 (31–45) mm for men and 35 (30–40) mm for women (*P* = 0.000002, *t*-test). The allowance angle corresponding to medialization of screw sites for the average retroacetabular angle and tangent for both male and female patients is represented in Figure 4.

**Figure 5:**
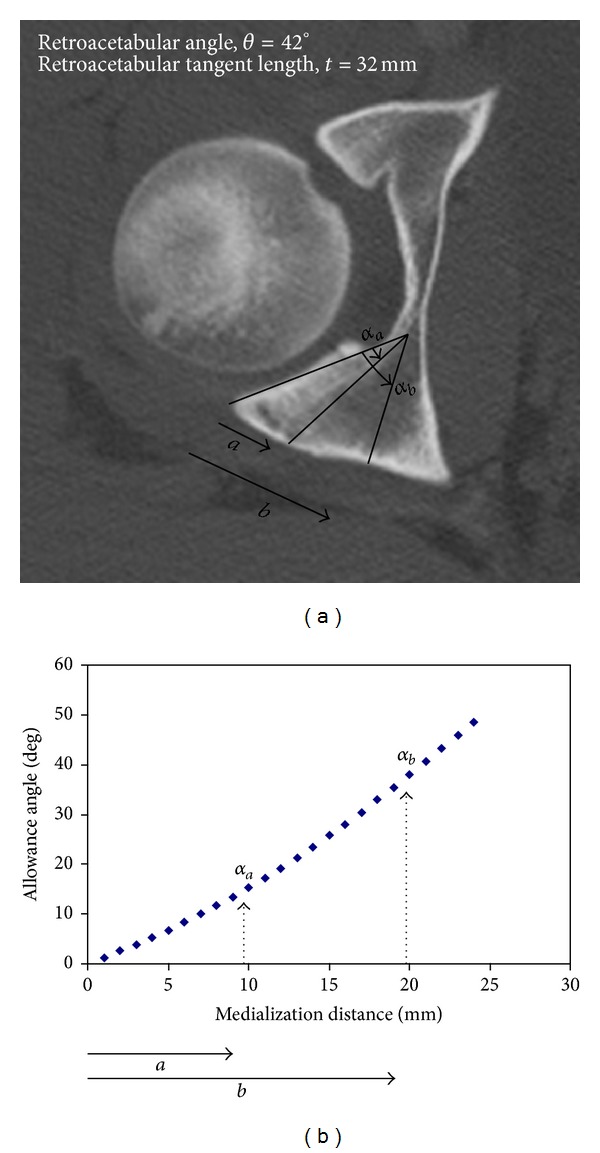
(a) Pelvis CT scan of a patient with acetabular fracture who has a retroacetabular angle of 42 degrees and a retroacetabular tangent length of 32 mm. The contralateral normal acetabulum is used for preoperative angle assessment. The medialization distances *a* and *b* correspond to allowance angles *α*_*a*_ and *α*_*b*_. (b) The increment in the magnitude of the allowance angle in relation to the medialization distance for the same patient is graphically demonstrated by a curve plotted using a computer program for preoperative planning.

The software created can be used to provide an allowance angle from entry of the retroacetabular angle and the retroacetabular tangent as measured in the trauma pelvis CT scan. The allowance angle corresponding to each mm increment in medialization distance is thus calculated swiftly. An example for computing the allowance angles in a patient with retroacetabular angle of 42 degrees and retroacetabular tangent of 32 mm is shown in Table 1. A curve showing the relation between the medialization distance and the allowance angle is also plotted by the computer program (Figure 5).

## 4. Discussion

 Intraarticular screw placement is a recognized complication of acetabular surgery [7, 8]. Posterior wall fractures being the most commonly operated acetabular fractures necessitated the development of techniques for safe screw placement. Placing Kirschner (K) wires tangential to the articular surface under direct vision at the proximal and distal extent of the intact acetabular rim allows a fixed plane of reference [7]. Bosse advised to place screws in the posterior wall in the coronal plane perpendicular to the long axis of the body [10]. Ebraheim et al. demonstrated in a cadaver study the danger zone of the posterior wall of the acetabulum to be widest at the midacetabular region [9]. They described an angle of medial angulation from the perpendicular to the posterior wall for a safe screw path starting one cm medial to the posterior acetabular margin. They pioneered quantification of the amount of angulation needed to safely place screws in the danger zone of the acetabulum. We think that the angle we chose to measure “the retroacetabular angle” is anatomical as it is the angle of the posterior margin of the acetabulum. It not an arbitrary angle assumed some distance along the posterior wall, so it is easier to measure. The measurements are not dependent on other parameters, for example, the perpendicular to the posterior acetabular wall as in case of the angle described by Ebraheim et al. [9], the determination of which may compromise the accuracy. The addition of the allowance angle to the retroacetabular angle provides the safe angle for screw placement for every one millimeter along the whole posterior wall and column and not merely at fixed points on the posterior wall (Table 1). 

The angle for safe screw placement which is measured, preoperatively, is the angle between the screw and the retroacetabular surface. The latter is represented by a straight line *S* drawn on axial CT scan (Figures 1 and 3). However, the retroacetabular surface, in reality, is slightly curved, so it forms a double slope in relation to the straight line *S*, used for preoperative planning. This makes the screw angles measured in the lateral and more dangerous zone of the posterior wall slightly smaller than the maximum allowed. On the other hand, the angles measured in the medial part of the posterior wall are slightly bigger. However, the medial region is generally safe. This shows the need for an accurate drill guide that defines the planned angles accurately and at the same time nullifies the slight errors produced by the anatomical irregularity of the posterior acetabular wall. The data provided by this study could be used as a theoretical basis for manufacturing such a drill guide for safe screw placement through the posterior acetabular wall. 

This study was based on CTs of a large consecutive series of acetabular fracture patients that included both men and women of wide age range. Consequently, our results would be more representative of the population vulnerable to acetabular fractures. We think that the latter goal would not be achieved by conducting the same study on cadavers. Cadavers are less available than CT scans, so much fewer would be used. Most cadavers are of the geriatric population with advanced osteoarthritis and would not truly represent acetabular fracture patients. Considering that previous studies specifically dealing with placing screws in the posterior acetabular wall were based on cadavers [9], we opted to replace the latter by CT scans in our study. Similarly, three-dimensional CT scan models were used by Attias et al. to evaluate the intraosseous space available for percutaneous screw fixation for acetabular fracture fixation [12]. Additionally, various preoperative planning tools based on CT scan have been provided [11, 13–15]. However, they require sophisticated equipments and expensive computer software. They require lengthy preoperative procedures which might necessitate the support of computer engineers. The tool we provide, specifically, deals with posterior acetabular fractures which are commonly managed by general orthopaedic trauma surgeons. It entails simple measurements which could be done in few minutes. It is based on axial CT without any technical demand for complex reconstruction or engineering support. We were similar to Brown et al. [11] in using the contralateral normal pelvis as the basis for preoperative planning to avoid the process of image segmentation and the need for virtual fragment manipulation which is done by computer engineers [13–15].

The aim for measuring the retroacetabular angle in a series of patients is to get a crude guide for the safe angle of screw placement for surgeons who opt not to use the case-to-case assessment described above for their preoperative planning. We did our measurements at the middle of the danger zone of the posterior wall which proved to be the thinnest part in recent anatomical studies of the pelvis [5]. We think this would provide the safest possibilities for screw placement through the posterior wall.

The retroacetabular angle, as measured in CT cuts, is smaller than the anatomical ones which are measured in a plane perpendicular to the acetabular margin (Figure 2). The difference can be explained geometrically, by the similarity of the posterior acetabular wall to a three-sided prism where a plane perpendicular to the prism sides has a bigger apex angle than that of an oblique plane (Figure 2(b)).

The retroacetabular angle was significantly smaller in female patients. This theoretically makes them more vulnerable to intraarticular hardware penetration of the acetabulum. 

From the clinical point of view, case-to-case assessment, facilitated by our simple computer program can determine precisely the maximum safe angles of anterior inclination from the retroacetabular surface for screw placement in the posterior acetabular wall. The screws should be placed as perpendicular as possible to the fracture plane to allow maximum compression. This might be possible, depending on the fracture configuration, at an angle smaller than the maximum safe angle. However, the latter should not be exceeded.

To summarize, the posterior articular surface of the acetabulum makes an average (range) angle of 39 (33–47) degrees with the retroacetabular surface. Placing the screws at an equivalent angle of anterior inclination with the retroacetabular surface makes them parallel to the posterior articular surface, thus avoiding intraarticular penetration. With more medially placed screws the safe angle for screw, placement becomes larger. The increment (the allowance angle) can be calculated for every one mm of medialization, from the posterior acetabular margin, of screw site. All of the calculations are facilitated by a simple computer program.

## Supplementary Material

Computer software (Excel, Microsoft Office, version 2003,Microsoft, USA) spread sheet that can be used to calculate the allowance angles corresponding to variable retroacetabular angles and tangents for every one mm of medialization distance of screw site. It can be used by any surgeon pre or intraoperatively to determine proper angles for screw trajectory in the posterior acetabular wall.

## Figures and Tables

**Table 1:** Example of the computed allowance angles for each one mm of medialization of screw site in the posterior acetabular wall for a patient with a retroacetabular angle of 42 degrees and a retroacetabular tangent of 32 mm as it appears in our computer program.

Retoacetabular tangent length (*t*) = 32 mm	Retroacetabular angle (*θ*) = 42 degrees
Medialization distance (mm)	Allowance angles (degrees)
0.01	0.012
1.0	1.24
2.0	2.52
3.0	3.87
4.0	5.28
5.0	6.76
6.0	8.31
7.0	9.93
8.0	11.63
9.0	13.40
10.0	15.25
11.0	17.19
12.0	19.20
13.0	21.30
14.0	23.47
15.0	25.73
16.0	28.05
17.0	30.45
18.0	32.92
19.0	35.44
20.0	38.01
21.0	40.63
22.0	43.27
23.0	45.94
24.0	48.61

## References

[1] Guyton JL, Crockarell JR, Canale ST (2003). Fractures of the acetabulum and pelvis. *Campbell’s Operative Orthopaedics*.

[2] Heeg M, Klasen HJ, Visser JD (1990). Operative treatment for acetabular fractures. *Journal of Bone and Joint Surgery B*.

[3] Goulet JA, Rouleau JP, Mason DJ, Goldstein SA (1994). Comminuted fractures of the posterior wall of the acetabulum. A biomechanical evaluation of fixation methods. *Journal of Bone and Joint Surgery A*.

[4] Matta JM, Letournel E, Browner BD (1986). Surgical management of acetabular fractures. *Instructional Course Lectures*.

[5] Shahulhameed A, Roberts CS, Pomeroy CL, Acland RD, Giannoudis PV (2010). Mapping the columns of the acetabulum-Implications for percutaneous fixation. *Injury*.

[6] Mast J, Jakob R, Ganz R (1989). *Planning and Reduction Technique in Fracture Surgery*.

[7] Letournel E, Judet R, Elson RA (1992). *Fractures of the Acetabulum*.

[8] Letournel E (1980). Acetabulum fractures: classification and management. *Clinical Orthopaedics and Related Research*.

[9] Ebraheim NA, Waldrop J, Yeasting RA, Jackson WT (1992). Danger zone of the acetabulum. *Journal of Orthopaedic Trauma*.

[10] Bosse MJ (1991). Posterior acetabular wall fractures: a technique for screw placement. *Journal of Orthopaedic Trauma*.

[11] Brown GA, Milner B, Firoozbakhsh K (2002). Application of computer-generated stereolithography and interpositioning template in acetabular fractures: a report of eight cases. *Journal of Orthopaedic Trauma*.

[12] Attias N, Lindsey RW, Starr AJ, Borer D, Bridges K, Hipp JA (2005). The use of a virtual three-dimensional model to evaluate the intraosseous space available for percutaneous screw fixation of acetabular fractures. *Journal of Bone and Joint Surgery B*.

[13] Cimerman M, Kristan A (2007). Preoperative planning in pelvic and acetabular surgery: the value of advanced computerised planning modules. *Injury*.

[14] Citak M, Gardner MJ, Kendoff D (2008). Virtual 3D planning of acetabular fracture reduction. *Journal of Orthopaedic Research*.

[15] Fornaro J, Kee M, Harders M, Marincek1 B, Székely G, Frauenfelder T (2010). An interactive surgical planning tool for acetabular fractures: initial results. *Journal of Orthopaedic Surgery and Research*.

